# Incidental Detection of a Pulmonary Valve Thrombus in a 60-Year-Old Woman: A Case Report

**DOI:** 10.7759/cureus.119645

**Published:** 2026-11-14

**Authors:** Abhishek Singh, Naveen Sharma, Vinay Bhatia

**Affiliations:** 1 Cardiology, Maidstone and Tunbridge Wells NHS Trust, Dartford, GBR; 2 Radiology, Maidstone and Tunbridge Wells NHS Trust, Tunbridge Wells, GBR; 3 Cardiology, Maidstone and Tunbridge Wells NHS Trust, Tunbridge Wells, GBR

**Keywords:** cardiac magnetic resonance, ct pulmonary angiogram (ctpa), echocardiography, intracardiac thrombus, pulmonary valve mass, pulmonary valve thrombus

## Abstract

Pulmonary valve thrombus is a rare cardiac finding, particularly when identified incidentally in a patient without established venous thromboembolism or infective endocarditis. We report the case of a 60-year-old woman who presented with atypical chest discomfort and exertional dyspnoea. She described a sensation of being unable to take an adequate breath, while her husband had noticed increased breathing effort during routine walking. She had no previous history of ischemic heart disease or cerebrovascular disease and was a lifelong non-smoker. Cardiovascular examination was unremarkable, with normal blood pressure and oxygen saturation. Transthoracic echocardiography initially demonstrated no significant structural cardiac abnormalities or valvular pathology. CT coronary angiography, performed to investigate her symptoms, demonstrated no flow-limiting coronary artery disease but incidentally identified a filling defect associated with the pulmonary valve. Dedicated CT pulmonary angiography excluded pulmonary embolism but confirmed the pulmonary valve lesion. Cardiac MRI demonstrated a mass associated with the pulmonary valve, with appearances most consistent with thrombus. There was no clinical, laboratory, or imaging evidence of infective endocarditis. The patient was treated with warfarin anticoagulation. Follow-up CT pulmonary angiography after three months demonstrated complete resolution of the pulmonary valve lesion, supporting the diagnosis of thrombus. This case highlights the importance of considering pulmonary valve thrombus in the differential diagnosis of an incidentally detected valvular mass and demonstrates the value of multimodality cardiac imaging in differentiating thrombus from infective vegetation and benign valvular tumours.

## Introduction

Intracardiac thrombus is an important complication of a range of cardiovascular and systemic disorders. Thrombus formation is generally understood in the context of abnormalities in blood flow, endothelial injury, and hypercoagulability, traditionally described by Virchow’s triad [1]. In clinical practice, intracardiac thrombi are most frequently encountered in the left-sided chambers, particularly the left ventricle and left atrium or left atrial appendage. Common predisposing conditions include left ventricular systolic dysfunction, myocardial infarction, atrial fibrillation, ventricular aneurysm, cardiomyopathy, and other conditions associated with blood stasis or a pro-thrombotic state [2]. The location of intracardiac thrombus has important implications for both diagnosis and embolic risk.

Right-sided intracardiac thrombi are considerably less common than left-sided thrombi. They may occur in association with pulmonary embolism, deep venous thrombosis, malignancy, right ventricular dysfunction, intracardiac devices, central venous catheters, and other pro-thrombotic conditions [3,4]. Mobile right-heart thrombi, sometimes referred to as thrombi in transit, are particularly important because they are frequently associated with pulmonary embolism and may carry a substantial risk of clinical deterioration and mortality [3,4]. Management of right-heart thrombi remains challenging because evidence is derived predominantly from observational studies, case series, and expert opinion, with therapeutic options including anticoagulation, systemic thrombolysis, catheter-based intervention, and surgical thrombectomy in selected patients [3,4].

Thrombus specifically involving the native pulmonary valve is exceptionally uncommon. The pulmonary valve is exposed to high-flow conditions and is therefore an unusual site for thrombus formation in the absence of structural valve disease, pulmonary hypertension, pulmonary embolism, intracardiac instrumentation, or another pro-thrombotic substrate. Published evidence concerning pulmonary valve thrombosis is largely limited to individual case reports. Rigopoulos et al. described a mobile pulmonary valve thrombus in a patient with chronic thromboembolic pulmonary hypertension and pulmonary embolism, illustrating the association between pulmonary vascular disease and pulmonary valve thrombus [5]. However, isolated pulmonary valve thrombus in a patient without pulmonary embolism or pulmonary hypertension appears to be particularly rare.

An unexpected pulmonary valve mass presents a diagnostic challenge because thrombus may closely resemble other valvular lesions on imaging. The differential diagnosis includes infective vegetation, papillary fibroelastoma, Lambl’s excrescences, and, less commonly, other benign or malignant cardiac masses [6-9]. Infective endocarditis remains an important consideration whenever a valvular mass is identified, although the diagnosis requires integration of clinical, microbiological, and imaging findings rather than reliance on the presence of a mass alone [10,11]. Conversely, papillary fibroelastoma is a benign valvular tumour that is typically small, mobile, and pedunculated and may be incidentally discovered during cardiac imaging. Although most fibroelastomas arise from left-sided valves, pulmonary valve involvement has been reported and may occasionally present with dyspnoea, pulmonary embolic phenomena, or right ventricular outflow tract obstruction [6-9].

The increasing use of cardiovascular CT, cardiac magnetic resonance (CMR), and advanced echocardiography has expanded the ability to characterise intracardiac and valvular masses non-invasively. Echocardiography remains a fundamental first-line modality because of its accessibility and ability to assess mobility, attachment, haemodynamic consequences, and valve function. CT provides high spatial resolution and may be particularly useful for defining the relationship between a lesion and the pulmonary valve or pulmonary arterial tree. CMR provides additional tissue characterisation and can help distinguish thrombus from vascularised tumour through assessment of perfusion and late gadolinium enhancement characteristics [12-14]. Current literature therefore supports a multimodality approach when the nature of an intracardiac mass remains uncertain.

We report the case of an incidentally detected pulmonary valve thrombus in a 60-year-old woman investigated for atypical chest discomfort and exertional dyspnoea. The lesion was identified on CT coronary angiography, further evaluated using dedicated CT pulmonary angiography and CMR, and subsequently demonstrated complete radiological resolution following anticoagulation. The case is unusual because there was no pulmonary embolism, pulmonary hypertension, right ventricular dysfunction, intracardiac device, or other clear predisposing cardiac abnormality. It highlights the diagnostic difficulty of an unexpected pulmonary valve mass and illustrates the potential value of multimodality imaging and therapeutic response in establishing a diagnosis when histopathological confirmation is unavailable.

## Case presentation

A 60-year-old woman was reviewed following episodes of atypical chest discomfort and exertional dyspnoea. She described a sensation of being unable to get sufficient air into her lungs. Her husband had additionally noticed that she appeared to be breathing more heavily when walking around town. She reported an element of chest discomfort but found it difficult to characterise the nature, location, or precipitating factors of this discomfort.

She was a lifelong non-smoker and reported alcohol consumption of up to 16 units per week. There was no previous history of ischaemic heart disease or cerebrovascular events. Her family history was notable for her father having undergone coronary artery bypass surgery at a relatively young age, followed subsequently by pacemaker implantation.

On clinical assessment, her blood pressure was 132/67 mmHg in the right arm and her oxygen saturation was 98% on room air. Her weight was 74 kg, corresponding to a body mass index of approximately 28 kg/m². She was comfortable at rest. The jugular venous pressure was not visibly elevated. Cardiac examination revealed normal heart sounds without an audible murmur. There was normal air entry bilaterally and no significant peripheral oedema. Table 1 presents laboratory investigation findings.

**Table 1:** Summary of laboratory investigations performed during the patient’s assessment. Values are presented with the corresponding laboratory reference ranges where applicable. HbA1c: glycated haemoglobin; HDL: high-density lipoprotein; LDL: low-density lipoprotein; TSH: thyroid-stimulating hormone; CRP: C-reactive protein

Investigation	Result	Reference range
Haemoglobin	138 g/L	115–165 g/L
White cell count	4.5 × 10⁹/L	4.0–11.0 × 10⁹/L
HbA1c	36 mmol/mol	20–41 mmol/mol
Total cholesterol	5.5 mmol/L	<5.0 mmol/L
Triglycerides	0.6 mmol/L	<1.7 mmol/L
HDL cholesterol	2.3 mmol/L	>1.0 mmol/L
LDL cholesterol	2.9 mmol/L	<3.0 mmol/L
Renal function	Normal	Laboratory reference range
Liver function	Normal	Laboratory reference range
TSH	Normal	0.4–4.0 mIU/L
CRP	<1 mg/L	<5 mg/L
Blood cultures (3 sets)	Negative	Negative

Given her symptoms, transthoracic echocardiography (TTE) was performed. Transthoracic echocardiographic views were suboptimal due to the patient’s body habitus, limiting detailed visualisation of the cardiac valves, particularly the pulmonary valve. No significant valvular abnormality was identified in the available views. Biventricular size and systolic function were preserved. The peak pulmonary valve velocity was 0.7 m/s, corresponding to a peak pressure gradient of 2.1 mmHg. Pulmonary acceleration time was normal, and the estimated pulmonary artery systolic pressure was 19 mmHg, with no echocardiographic evidence suggestive of pulmonary hypertension. Overall, it showed no significant structural abnormalities or cardiac valve pathology.

CT coronary angiography was subsequently performed to exclude significant coronary artery disease. This demonstrated no flow-limiting coronary artery disease. However, an incidental filling defect associated with the pulmonary valve was identified and reported as possibly representing a pulmonary valve thrombus.

Due to the CT findings, a comprehensive history was obtained from the patient, particularly to look for provoking or risk factors causing thrombus. She had no history of previous thromboembolism. Had not undergone recent surgery or immobilisation. She had no history of previous malignancy and had not received any hormonal treatment in the past.

A dedicated CT pulmonary angiogram was therefore performed, which excluded pulmonary embolism but confirmed the filling defect associated with the pulmonary valve (Figure 1).

**Figure 1:**
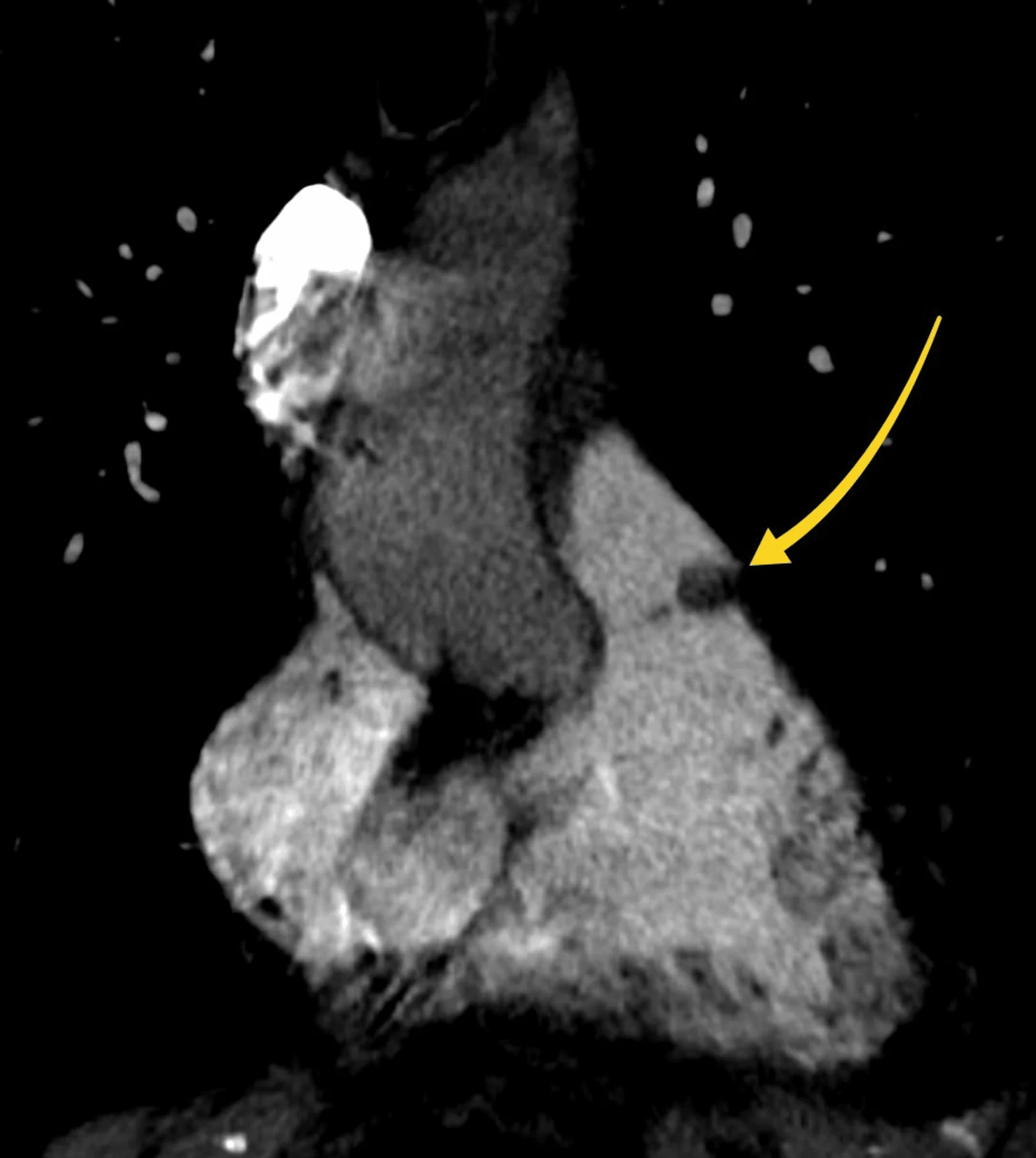
Initial post-contrast CT showing a view of the right ventricle and a filling defect on the pulmonary valve.

Following the incidental detection of a pulmonary valve filling defect on CT imaging, the case was discussed at the multidisciplinary team meeting. The consensus recommendation was to proceed with CMR imaging for further characterisation of the lesion and to aid diagnostic assessment.

CMR demonstrated a persistent 10 mm solid ovoid lesion involving the pulmonary valve, unchanged compared with previous CT imaging. The lesion exhibited intermediate signal intensity on cine sequences without abnormal enhancement on delayed gadolinium imaging, with overall appearances favouring thrombus. Biventricular morphology and systolic function were preserved, with a left ventricular ejection fraction of 70% and a right ventricular ejection fraction RVEF of 52%. There was mild biatrial dilatation, but no evidence of myocardial infarction, infiltration, or fibrosis. The absence of delayed gadolinium enhancement supported the diagnosis of thrombus over an enhancing cardiac mass, although it is not independently diagnostic (Figure 2).

**Figure 2:**
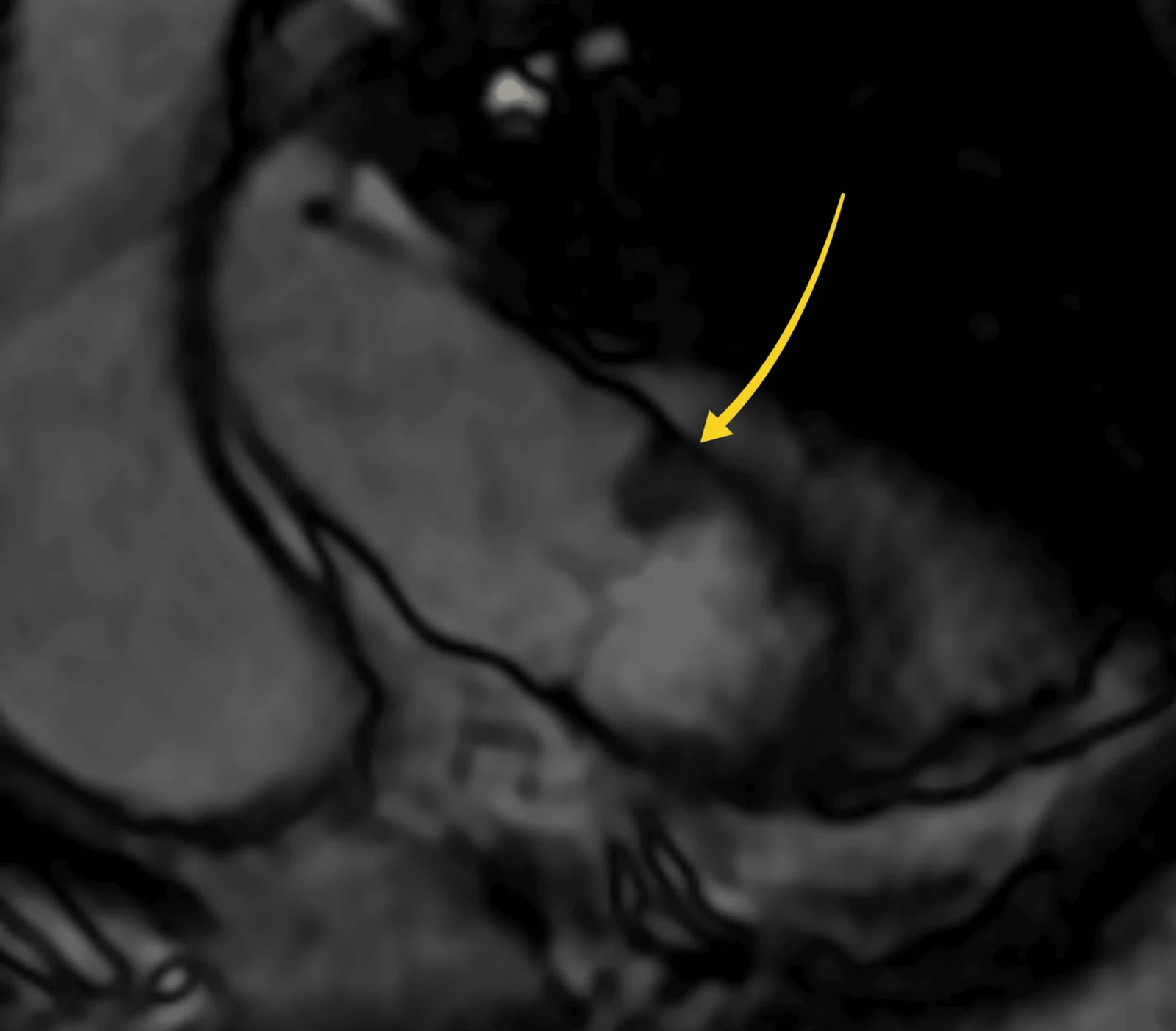
Still image from RVOT cine view of cardiac MRI confirming a lesion on the pulmonary valve. RVOT: right ventricular outflow tract

The unexpected pulmonary valve mass prompted consideration of several differential diagnoses, including thrombus, infective vegetation, and benign valvular tumour, particularly papillary fibroelastoma [4-6].

There was no clinical evidence of infective endocarditis. The patient was afebrile, inflammatory markers were normal, and blood cultures were negative. Three sets of blood cultures were obtained to investigate the possibility of infective endocarditis. In view of the absence of fever, peripheral stigmata of infective endocarditis on clinical examination, or biochemical evidence of infection or systemic inflammation, antibiotics were not commenced before blood culture collection. There was no evidence of pulmonary embolism clinically or on CT pulmonary angiography. Following multidisciplinary review of the clinical findings and multimodality imaging, the pulmonary valve lesion was considered most consistent with thrombus.

The patient was commenced on warfarin anticoagulation with regular international normalised ratio (INR) monitoring, targeting an INR of 2-3. The patient was continued on anticoagulation for at least three months. An interval CT pulmonary angiogram was planned because CT pulmonary angiography had provided the clearest visualisation of the lesion at presentation. The plan was to consider cardiothoracic surgical assessment if the lesion persisted.

After three months of anticoagulation, repeat CT pulmonary angiography demonstrated complete resolution of the previously identified pulmonary valve filling defect. This radiological resolution following anticoagulation provided additional support for the diagnosis of thrombus (Figure 3).

**Figure 3:**
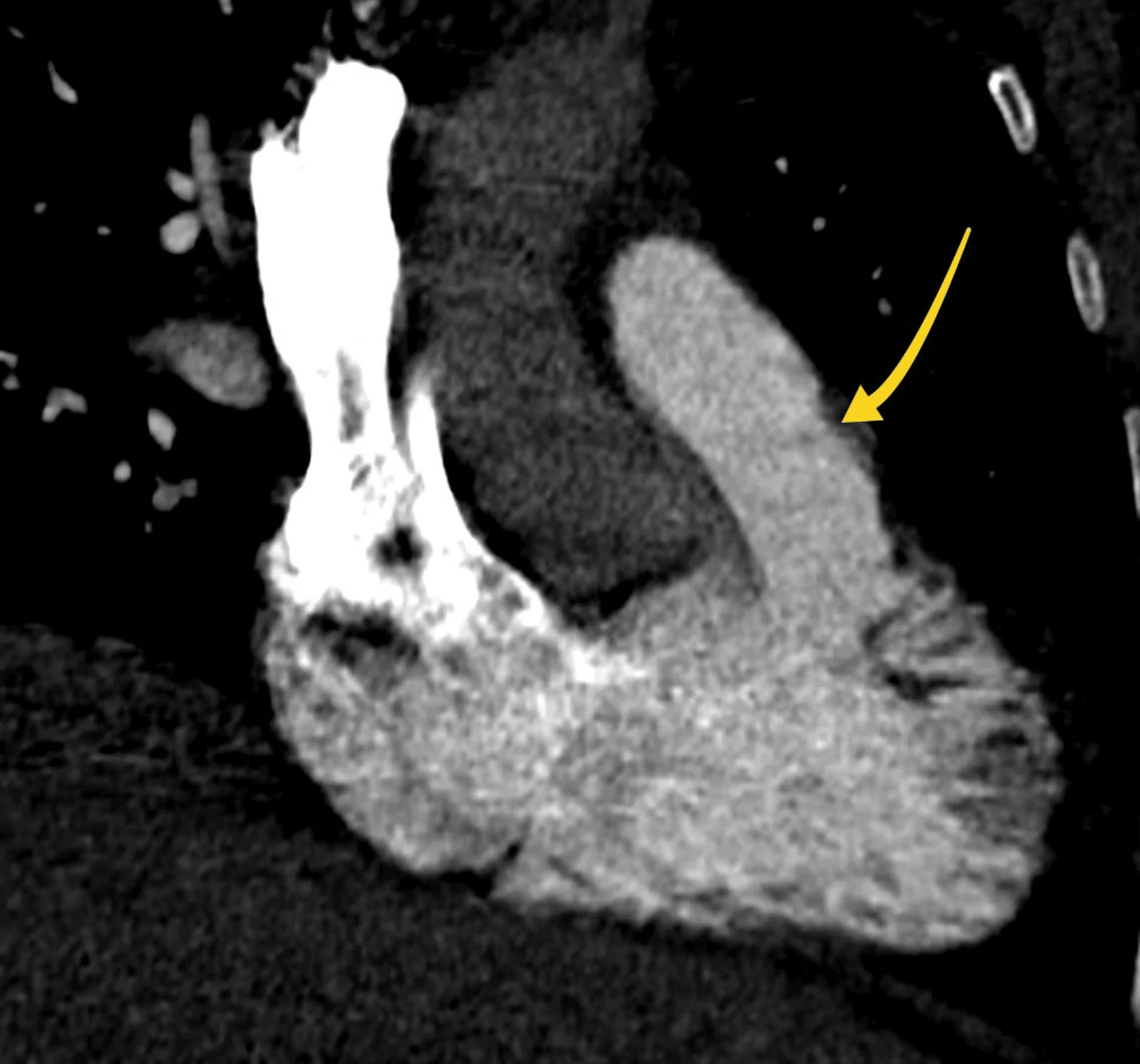
Repeat post-contrast-enhanced CT pulmonary angiography following treatment showing resolution of the previously identified filling defect.

Although the pulmonary valve lesion was initially detected incidentally on CT rather than TTE and the initial TTE failed to demonstrate the lesion, with visualisation limited by the patient’s body habitus, a repeat transoesophageal echocardiogram (TOE) was therefore performed following four months of warfarin anticoagulation. The available, albeit limited, views showed no convincing evidence of a residual mass or thrombus involving the pulmonary valve or right ventricular outflow tract. No thrombus was identified in the left atrial appendage, and there was no echocardiographic evidence of infective vegetations, abscess, or fistula formation. Overall, with limited visualisation, the TOE findings were reassuring, with no obvious residual lesion identified.

She was reviewed subsequently in cardiology clinic follow-up where she was found to have complete resolution of her atypical chest discomfort and exertional dyspnoea. She remained clinically stable, with no further episodes of chest pain, dyspnoea, or other persistent symptoms. She was also referred to the haematology team for a thrombophilia screen to investigate any underlying predisposition to thrombus formation.

## Discussion

Pulmonary valve thrombus is an exceptionally rare form of intracardiac thrombosis. Intracardiac thrombi more commonly occur in the left-sided chambers, particularly in association with ventricular dysfunction, myocardial infarction, atrial fibrillation, and other conditions causing blood stasis [1,2]. Right-heart thrombi are less common and are usually associated with pulmonary embolism, deep venous thrombosis, malignancy, right ventricular dysfunction, or intravascular devices [3,4]. The present case is unusual because the pulmonary valve thrombus occurred without pulmonary embolism, pulmonary hypertension, right ventricular dysfunction, or an intracardiac device.

The mechanism of thrombus formation in this patient is uncertain. Virchow’s triad describes the contribution of abnormal blood flow, endothelial injury, and hypercoagulability to thrombogenesis [1]. Although local flow disturbance or endothelial injury at the pulmonary valve may have contributed, no structural valve abnormality or systemic provoking factor was identified. Previously reported pulmonary valve thrombus has occurred in association with pulmonary vascular disease. Rigopoulos et al. described a mobile pulmonary valve thrombus in a patient with chronic thromboembolic pulmonary hypertension and pulmonary embolism [5]. In contrast, our patient had no evidence of pulmonary vascular disease, making this an unusual presentation of isolated pulmonary valve thrombosis.

An important diagnostic challenge is distinguishing thrombus from other pulmonary valve masses. Infective endocarditis should be considered whenever a valvular mass is detected, with diagnosis based on clinical findings, blood cultures, and cardiac imaging rather than the presence of a mass alone [10,11]. In this case, the absence of fever, normal inflammatory markers, negative blood cultures, absence of a new murmur, and lack of valvular destruction made infective endocarditis unlikely.

Papillary fibroelastoma is another important differential diagnosis. These benign valvular tumours are usually small, mobile, and pedunculated. Pulmonary valve involvement is rare, although systematic review and subsequent case reports have documented incidental pulmonary valve fibroelastomas and presentations including dyspnoea or embolic complications [6-9]. Differentiation from thrombus may be difficult on a single imaging modality, particularly when histological confirmation is unavailable.

Multimodality imaging was therefore central to the diagnosis. Echocardiography provided assessment of cardiac structure and valve function; however, atypically in our patient, it was challenging with limited views. With the views available, it was found to be normal, showing no significant structural abnormalities or cardiac valve pathology, while incidentally, CT demonstrated the pulmonary valve filling defect and excluded pulmonary embolism. CMR provided additional tissue characterisation and supported thrombus as the most likely diagnosis. CMR is particularly useful in differentiating thrombus from vascularised cardiac masses through assessment of signal characteristics, perfusion, and enhancement [12-14]. The complementary roles of echocardiography, CT, and CMR are increasingly recognised in the assessment of intracardiac masses [13,14].

The strongest evidence supporting the diagnosis in this case was the complete disappearance of the lesion following anticoagulation. Although therapeutic response cannot provide histological confirmation, resolution of a discrete valvular mass after anticoagulation strongly favours thrombus over a persistent neoplasm such as fibroelastoma. The combination of multimodality imaging, absence of infective features, and complete radiological resolution therefore provides a compelling clinical diagnosis.

There is no established treatment strategy specifically for isolated pulmonary valve thrombus. Management of right-heart thrombi may include anticoagulation, thrombolysis, surgical thrombectomy, or catheter-based intervention, depending on thrombus characteristics and clinical status [3,4]. Patients with right-heart thrombus and pulmonary embolism may require more aggressive treatment, whereas stable patients without pulmonary embolism may be managed conservatively [3,4,15]. In our patient, the absence of pulmonary embolism or haemodynamic compromise supported anticoagulation with warfarin. Complete resolution after three months allowed surgical intervention to be avoided.

The clinical significance of the thrombus in relation to the presenting symptoms remains uncertain. Although her dyspnoea and atypical chest discomfort improved following treatment, there was no pulmonary hypertension, right ventricular dysfunction, or significant pulmonary valve obstruction to establish a clear physiological mechanism. The lesion may therefore have been incidental.

This case has several limitations. Histopathological confirmation was unavailable because the lesion resolved with anticoagulation. In addition, no definite provoking factor was identified, and a causal relationship between the thrombus and the patient’s symptoms cannot be established. Nevertheless, the combination of clinical assessment, negative infective investigations, multimodality imaging, and complete resolution with anticoagulation provides strong supportive evidence for isolated pulmonary valve thrombus.

Overall, this case highlights that pulmonary valve thrombus should be considered in the differential diagnosis of an incidentally detected pulmonary valve mass, even in the absence of pulmonary embolism or conventional thrombotic risk factors. A multimodality imaging approach can help distinguish thrombus from infective vegetation and papillary fibroelastoma, while interval imaging can provide important diagnostic and therapeutic information.

## Conclusions

Pulmonary valve thrombus is an exceptionally rare but important differential diagnosis when a pulmonary valve mass is identified. Although right-sided intracardiac thrombi are frequently associated with pulmonary embolism and other recognised thrombotic conditions, pulmonary valve thrombus may rarely occur without pulmonary embolism, pulmonary hypertension, right ventricular dysfunction, or an obvious provoking factor. A structured assessment incorporating clinical findings, laboratory investigations, echocardiography, assessment for pulmonary embolism, and appropriate multimodality imaging can help distinguish thrombus from important mimics such as infective vegetation and papillary fibroelastoma. In the absence of histopathological confirmation, a reasonable management approach is to initiate anticoagulation when clinical and multimodality imaging findings favour thrombus. Although subsequent resolution of the lesion and improvement in clinical symptoms following anticoagulation provide supportive evidence for a thrombotic aetiology, the absence of histopathological examination means that a definitive diagnosis cannot be established.
